# Thermographic analysis of infants faces during breastfeeding before and after lingual frenotomy

**DOI:** 10.1590/2317-1782/e20240396en

**Published:** 2026-02-06

**Authors:** Midiane Gomes da Silva, Erissandra Gomes, Danielle Pereira de Lima, Paula Fernanda Rocha de Assis Santana, Ana Paula Alves Figueiredo Lima, Aline Natallia Simões de Almeida, Sara Loureiro de Souza Ferreira, Daniele Andrade da Cunha, Roberta de Castro Martinelli, Denise Sabbagh Haddad, Marcos Leal Brioschi, Hilton Justino da Silva

**Affiliations:** 1 Universidade Federal de Pernambuco – UFPE - Recife (PE), Brasil.; 2 Universidade Federal do Rio Grande do Sul – UFRGS - Porto Alegre (RS), Brasil.; 3 Universidade de São Paulo – USP - São Paulo (SP), Brasil.

**Keywords:** Breast Feeding, Ankyloglossia, Surgery, Thermography, Suction

## Abstract

**Purpose:**

To analyze surface skin temperature with infrared thermography (IRT) in the regions of the temporal, masseter, and buccinator muscles during breastfeeding before and after LF.

**Methods:**

Non-randomized clinical trial in 40 infants diagnosed with ankyloglossia. The lingual frenulum was assessed with the Neonatal Tongue Screening Test, breastfeeding was assessed with a protocol and pain scale, and the regions of interest were qualitatively and quantitatively assessed with IRT. Two independent evaluators analyzed the data.

**Results:**

There were post-LF improvements in the functional-anatomical tongue assessment (p < 0.001), breastfeeding pain scale (p < 0.001), and breastfeeding assessment regarding the mother’s general aspect (p < 0.001), breast pain (p = 0.03), and suction (p < 0.001). IRT data after LF showed a qualitative increase in temperature in the regions of the temporal and masseter muscles. There was no difference in the region of the buccinator muscle.

**Conclusion:**

LF impacts the surface skin temperature in the regions of mandibular levator muscles during breastfeeding.

## INTRODUCTION

Ankyloglossia is a congenital abnormality of the lingual frenulum that limits tongue movements and occurs when soft tissues that should have undergone apoptosis during embryonic development remain on the lower surface of the tongue^(1,2)^. Ankyloglossia is hereditary and occurs more commonly in males, in a ratio of 3:1^(2,3)^. There are still gaps in knowledge and evidence about the diagnosis, management and treatment of ankyloglossia, although functional limitations and impaired breastfeeding have been described in the literature^(4,5)^.

Babies with ankyloglossia may have difficulties latching on and sucking. The inefficient protrusion of the tongue over the lower alveolar ridge during sucking makes it difficult to inhibit the bite reflex, lip closure, grasping and muscle contraction in a distal to proximal direction. This creates an inadequate intraoral vacuum, which is necessary to extract breast milk from the lactiferous ducts. These difficulties can result in sore and/or cracked nipples, engorged breasts and other problems that can lead to early weaning. Surgical intervention called lingual frenotomy (LF) is indicated in cases of negative impact on breastfeeding^(1)^.

There is no gold standard for diagnosing ankyloglossia. Thus, functional-anatomical assessments are recommended^(6,7)^, complemented by breastfeeding assessments based on protocols with standardized measurements^(8)^. However, protocols that specifically assess sucking during breastfeeding are lacking. Although studies have described quantitative assessments of sucking patterns using video recordings, magnetic resonance imaging, ultrasound and electromyography, some authors have discussed the importance of using new technologies^(9-14)^.

Infrared Thermography (IRT) is a technology that captures the thermal distribution emitted by infrared waves throughout the human body, according to changes in body temperature related to superficial blood flow. It is a non-invasive, painless, fast technique with no contraindications or side effects that diagnoses physiological dysfunctions, evaluates and quantifies temperature variations, including in the craniofacial region^(15,16)^. The human face has temperature gradients, and physical quantities that quantitatively and qualitatively describe gradual and continuous changes in temperature. Thus, anatomical thermal points in the frontal and lateral views of the human face have been identified, mapped and quantified helping to diagnose and plan interventions for orofacial and cervical alterations^(17)^. The temperature gradients in the facial region may reflect underlying muscle activation, given that increased blood flow and thermogenic activity are associated with muscular effort^(18-20)^.

This study is justified by the interest in understanding the pattern of musculoskeletal activation on the face of infants during sucking. In breastfeeding, research has shown a characteristic temperature pattern in the breasts of breastfeeding women and its relationship with pathological aspects, which may be related to the baby's inadequate latch-on^(21,22)^.

The aim of analyzing the surface temperature of the skin of infants with IRT in the regions of the temporalis, masseter and buccinator muscles during breastfeeding before and after LF.

## METHODS

### Research design and study population

This study was designed as a nonrandomized clinical trial with a convenience sample of infants diagnosed with ankyloglossia and with an indication for LF. All infants had been previously evaluated and diagnosed with ankyloglossia by health professionals. The inclusion criteria were infants two to 30 days old, weighing 2,500 grams or more, on exclusive breastfeeding, and whose one-minute and five-minute Apgar scores were between seven and ten. Premature or twin infants and those with neurological or respiratory changes, cardiopathies, craniofacial deformities, or any other medical complication described by the physician were excluded from the research. Infants whose mothers had a medical diagnosis of breast condition or could not breastfeed were also excluded.

### Ethical considerations

This study was approved by the Human Research Ethics Committee, under evaluation report no. 5.520.664 and certificate no. CAEE 56736722.1.0000.5208, and was conducted according to the principles of the Declaration of Helsinki. The infants, mothers signed an informed consent form regarding themselves and as the ones responsible for the infants.

### Assessment

Ankyloglossia was diagnosed by a speech therapist using a functional-anatomical assessment of the lingual frenulum (Neonatal Tongue Screening Test)^(7)^, with the following items: lip posture at rest, tendency to position the tongue when crying, shape of the tip of the tongue raised when crying or during a lifting maneuver, frenulum thickness and fixation of the frenulum under the tongue and on the floor of the mouth. A score of seven or less was considered abnormal.

The speech therapist assessed breastfeeding using the Breastfeeding Assessment and Observation Form recommended by the World Health Organization (WHO) and the United Nations Children's Fund (UNICEF)^(23)^. This protocol has five categories of favorable and unfavorable behavior (suggestive of difficulties): mother's body and baby's body position, responses at the start of breastfeeding, breast condition, baby's position and grip and effective aspects of sucking, thus verifying the performance of the mother/baby dyad. In this study, when an item in each category suggested difficulties, it was classified as unfavorable behavior.

IRT was chosen as an evaluation technology because it is a non-invasive, painless, fast technique with no contraindications or side effects that diagnoses physiological dysfunctions, evaluates and quantifies temperature variations, including in the craniofacial region, and because it is a study population that requires less intervention and more sensitivity. IRT's validity in orofacial muscle analysis is described in literature^(18-20)^. The IRT images were obtained by the speech therapist using the protocol for IRT analysis of the face during breastfeeding suction^(24)^.

### Data collection

Data was collected at two different times for each baby: before LF and seven days after the surgical procedure. The baby's lingual frenulum was assessed by the speech therapist, using the anatomical-functional protocol, and if any alterations were found, the parents/guardians were instructed to carry out blood tests on their babies (complete blood count and coagulation and glycemia tests) if necessary for the surgical procedure. This was followed by the IRT.

Prior to the IRT assessment of the babies, their parents/guardians were instructed not to bathe the babies two hours before the procedure and not to put any adornment on their heads or perfume, cream or talcum powder. The mothers were instructed to wear comfortable, easy-to-remove clothes for the collection. The air conditioning was set at 22 to 24 ºC and the relative humidity was between 40 and 60%; the room temperature was stabilized with a digital thermohygrometer (AKSO - AK 28 new) for 15 minutes, the emissivity level was 0.98, the floor was thermally insulated and the room was lit with fluorescent lamps (cold).

**Figure 1:**
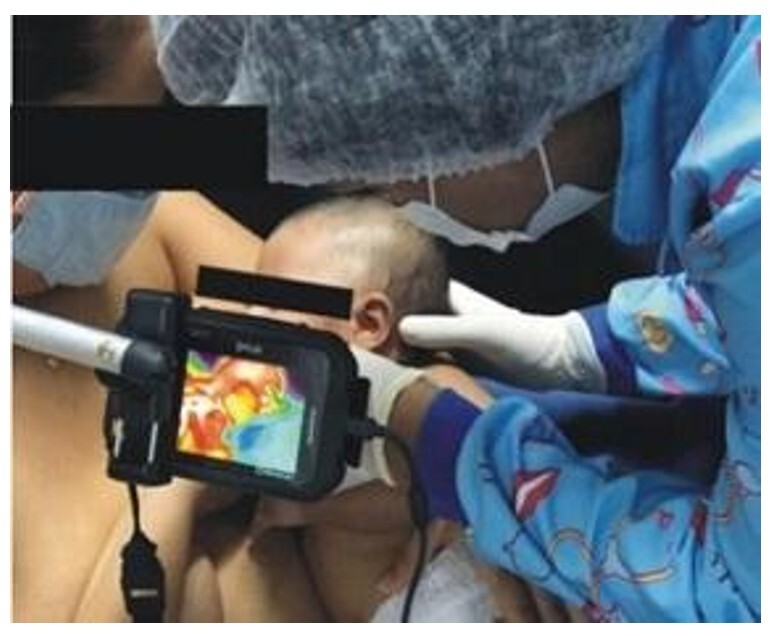
Adjusting the baby's head

**Figure 2:**
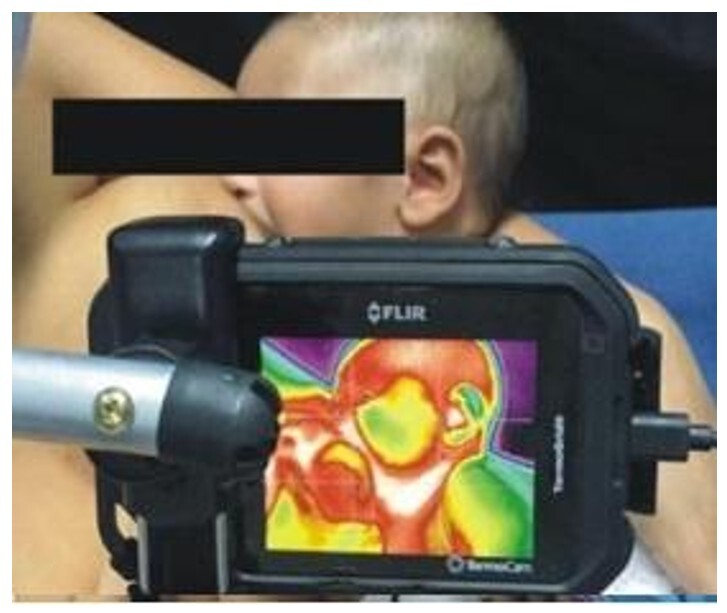
Capturing thermographic during breastfeeding

The mother and baby wore no clothing or adornments on their upper bodies, the mother was seated on a chair and cushion suitable for breastfeeding and was instructed to place the baby next to the breast, with the researcher helping to position the baby if necessary. At this point, IRT images were taken of the temporalis, masseter and buccinator muscles of one of the infant's hemifaces. The IRT camera - FLIR C2 (FLIR Inc., Santa Barbara, CA) was fixed to a tripod, tilted and positioned behind the chair, 15 centimeters away from the baby's face (Figure 1 and 2). The images were taken at the end of the 1st minute (1:59), between the 3rd and 4th minutes (3:30) and at the end of the 5th minute (4:59).

**Figure 3:**
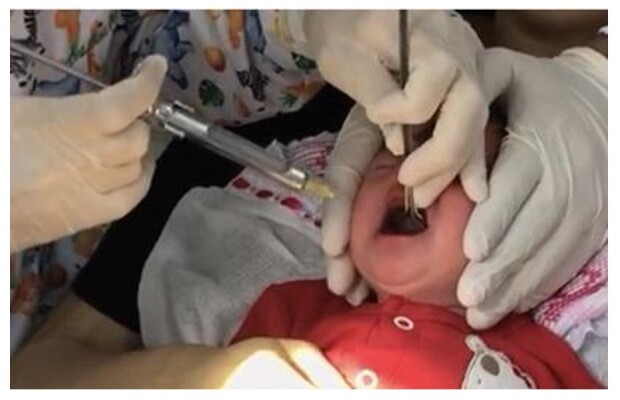
Lingual frenotomy

After the evaluations were completed, the infant underwent LF, which was performed by a dental surgeon specializing in pediatric dentistry. The mother was placed in the dental chair in the supine position and the baby on her lap in the same position. The dental surgeon applied infiltrative anesthesia and lifted the tongue with a sulcus guide to perform LF (Figure 3). After the procedure, the mother was instructed on the healing process.

Seven days after LF, the surgical procedure was evaluated by the same dental surgeon who performed the surgery and the lingual frenulum and breastfeeding were reassessed, with new IRT images being taken, following the same steps described above. During the assessment before LF and during the seven days afterwards, the mother was not given any guidance on breastfeeding and/or breast care in order to avoid bias during collection. After LF, if they still had difficulties, they were given support and instructions. Seven days was chosen to allow for primary wound healing while limiting the risk of external factors (e.g., guidance, therapy) that could confound muscle adaptation.

### Sample calculation

The sample size was calculated with WinPEPI (Windows Programs for Epidemiologists), version 11.65, based on a pilot study in ten infants. The calculation considered a 5% significance level, 80% power, an effect size of at least 0.5 standard deviations between the mean IRT results of the regions analyzed before and after LF, and a minimum 0.4 correlation coefficient between observations, obtaining a minimum total of 40 infants.

### Statistical analysis

**Figure 4:**
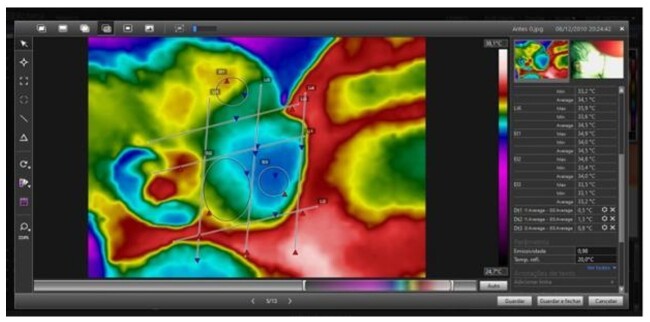
Quantitative analysis in FLIR Tools® software

IRT data were qualitatively analyzed by comparing images before and after LF with VisionFy^®^ software. Quantitative assessment was performed with FLIR Tools^®^ software, before and after LF in the region of interest (ROI) outlined on the face (Figure 4).

Two independent evaluators who had previous experience in assessing thermograms qualitatively and quantitatively analyzed the IRT images in two stages. The intrarater and interrater agreement was calculated with the Intraclass Correlation Coefficient (ICC). The mean temperature values for each ROI were considered, as extracted independently by the two evaluators.

Statistical analyses were performed in the Statistical Package for the Social Sciences (SPSS), v. 27.0 for Windows. Categorical variables were presented in relative and absolute frequencies, and the quantitative variables were presented in the median and amplitude of variation or the mean and standard deviation, according to data distribution. Asymmetrical variables were compared with the Wilcoxon test, and symmetrical ones, with Student’s t-test for paired samples. The McNemar chi-square test was used for the categorical variables. The significance level was set at 5%.

## RESULTS

The study assessed 40 infants, with a mean gestational age of 38.9±0.9 weeks, weighing 3,325±348 grams, 1-minute Apgar of 8.8±0.6, 5-minute Apgar of 9.7±0.5, and no neonatal complications other than ankyloglossia and breastfeeding difficulties. Only three (7.5%) out of the 27 (67.5%) who had difficulties before LF continued having them after LF. As for the mothers, 37 (92.5%) reported they had perceived improved sucking after LF.

Data on functional-anatomical lingual frenulum assessment were significantly different (p < 0.001) after LF in specific items and the total Neonatal Tongue Screening Test (Table 1).

**Table 1:** Neonatal Tongue Screening Test of infants before and after lingual frenotomy (LF) (n = 40)

	**Before LF**	**After LF**	**p-value**
**Lip posture at rest – n (%)**			
Closed lips (points=0)	32 (80)	36(90)
Parted lips (points=0)	4 (10)	4(10)
Open lips (points=1)	4 (10)	-
			
**Total of lip posture at rest – mean (min-max)**	0 (0-1)	0 (0-1)	0.206^*^
			
**Tongue position tendency when crying – n (%)**			
Tongue in the midline (points=0)	-	23(57.5)	
Lifted tongue (points=0)	2(5)	13(32.5)	
Tongue in the midline, lifting its sides (points=2)	27(67.5)	3(7.5)	
Lowered tongue tip, lifting its sides (points=2)	11(27.5)	1(2.5)	
			
**Total of the tongue position tendency when crying – mean (min-max)**			
	2 (0-2)	0 (0-2)	<0.001*
**Lifted tongue-tip shape when crying or in lifting maneuver – n (%)**			
Rounded (points=0)			
Slightly dipped tongue tip (points=2)	2(5)	12(30)	
Heart-shaped (points=3)	22(55)	28(70)	
	16(40)	-	
			
**Total of the lifted tongue-tip shape when crying or in lifting maneuver – mean (min-max)**			
	2 (0-3)	2 (0-2)	<0.001*
**Frenulum thickness – n (%)**			
Thin (points=0)	38(95)	24(60)	
Thick (points=2)	2(5)	16(40)	
		
**Total of the frenulum thickness – mean (min-max)**	0 (0-2)	0 (0-2)	<0.001#

* Wilcoxon test;

# Student’s t-test for paired samples;

**Caption:** mean±SD = mean+standard deviation; mean (min-max) = mean (minimum and maximum)

In breastfeeding, assessed with the said protocol, seven aspects had a significant difference before and after LF (Table 2).

**Table 2:** Breastfeeding of infants before and after lingual frenotomy (LF), observed using the Breastfeeding Assessment and Observation Form (n = 40)

	**Before LF**	**After LF**	**p-value**
	**n (%)**	**n (%)**	
**General**			
Mother			<0.001
Without signs of difficulties	25 (62.5)	39 (97.5)	0.250
With signs of difficulties	15 (37.5)	1 (2.5)	
Infant			
Without signs of difficulties	36 (90.0)	39 (97.5)	
With signs of difficulties	4 (10.0)	1 (2.5)	
**Breasts**			0.003
Without signs of difficulties	26 (65.0)	37 (92.5)	
With signs of difficulties	14 (35.0)	3 (7.5)	
**Infant’s position**			-
Without signs of difficulties	40 (100)	40 (100)	
With signs of difficulties	0 (0.0)	0 (0.0)	
**Infant’s latch**			-
Without signs of difficulties	0 (0.0)	0 (0.0)	
With signs of difficulties	40 (100)	40 (100)	
**Suction**			< 0.001
Without signs of difficulties	6 (15.0)	32 (80.0)	
With signs of difficulties	34 (85.0)	8 (20.0)	

McNemar test

Data on IRT assessment of the breastfeeding infants faces are shown in Tables 3 and 4. The qualitative analysis (Table 3) suggests that the temperature in the temporal and masseter regions increased in the majority of infants after LF at the 3rd minute of breastfeeding, indicating higher local muscular activation. Table 4 shows the quantitative analysis of the ROI averages (mean and standard deviation or minimum and maximum) at the three moments evaluated (1st, 3rd and 5th minutes), comparing before and after LF. The intra-rater ICC was above 0.9 in all qualitative and quantitative evaluation items, indicating an excellent level of agreement.

**Table 3:** Qualitative analysis of the ROI averages at the three moments evaluated (1st, 3rd and 5th minutes) of skin surface temperature in the temporal, masseter and buccinator muscle regions of breastfed infants before and after lingual frenotomy (LF) (n = 40)

**After vs. before LF Region of the temporal muscle**	**After vs. before LF Region of the masseter muscle**	**After vs. before LF Region of the buccinator muscle**
Increased (n = 24)	Increased (n = 26)	Increased (n = 19)
Decreased (n = 15)	Decreased (n = 14)	Decreased (n = 20)
Unchanged (n = 1)	Unchanged (n = 0)	Unchanged (n = 1)

**Table 4:** Mean and difference in skin surface temperature (in ºC) in the regions of interest (ROIs) of the temporal, masseter and buccinator muscles of breastfed infants before and after lingual frenotomy (LF) (n = 40)

	**Before LF** ºC	**After LF** ºC	**Difference (95% CI)** ºC	**p**
**1^st^ minute**				
Temporal ROIs - mean±SD	33.8±0.8	34±0.7	0.2 (-0.10 to 0.46)	0.203*
Masseter ROIs - mean±SD	33.4±0.9	33.4±0.8	0.0 (-0.34 to 0.32)	0.963*
Buccinator ROIs - mean±SD	32.6±1	32.6±0.9	0.0 (-0.36 to 0.40)	0.905*
Difference between temporal and masseter ROIs - mean (min-max)	0.5 (-0.7-1.4)	0.6 (-0.3-2.0)	-	0.056#
Difference between temporal and buccinator ROIs - mean (min-max)	1.1 (-0.1-2.8)	1.3 (0.3-3.0)	-	0.036^#^
Difference between masseter and buccinator ROIs - mean (min-max)	0.6 (0.1-1.5)	0.7 (0.1-1.5)	-	0.737^#^
**3^rd^ minute**				
Temporal ROIs - mean±SD	33.9±0.8	34±0.9	0.1 (-0.18 to 0.49)	0.347*
Masseter ROIs - mean±SD	33.4±1	33.5±1	0.1 (-0.29 to 0.44)	0.684*
Buccinator ROIs - mean±SD	32.8±1	32.8±1	0.0 (-0.39 to 0.43)	0.923*
Difference between temporal and masseter ROIs - mean (min-max)	0.3 (-0.6-1.4)	0.5 (-0.7-1.9)	-	0.052^#^
Difference between temporal and buccinator ROIs - mean (min-max)	1.1 (-0.4-2.7)	1.2 (-0.2-2.9)	-	0.393^#^
Difference between masseter and buccinator ROIs - mean (min-max)	0.6 (-0.1-1.6)	0.6 (-0.5-1.6)	-	0.975^#^
**5^th^ minute**				
Temporal ROIs - mean±SD	34±0.8	34.1±0.8	0.1 (-0.15 to 0.44)	0.334*
Masseter ROIs - mean±SD	33.5±1	33.5±0.9	0.0 (-0.28 to 0.39)	0.749*
Buccinator ROIs - mean±SD	32.8±1.1	32.9±1	0.0 (-0.41 to 0.44)	0.944*
Difference between temporal and masseter ROIs - mean (min-max)	0.4 (-0.3-1.5)	0.5 (-0.3-1.9)	-	0.952^#^
Difference between temporal and buccinator ROIs - mean (min-max)	1.1 (-0.1-2.7)	1.3 (-0.2-2.6)	-	0.068^#^
Difference between masseter and buccinator ROIs - mean (min-max)	0.5 (-0.1-1.5)	0.6 (0.1-1.5)	-	0.587^#^

* Student’s t-test for paired samples;

# Wilcoxon test;

**Caption:** mean±SD = mean+standard deviation; mean (min-max) = mean (minimum and maximum)

**Figure 5:**
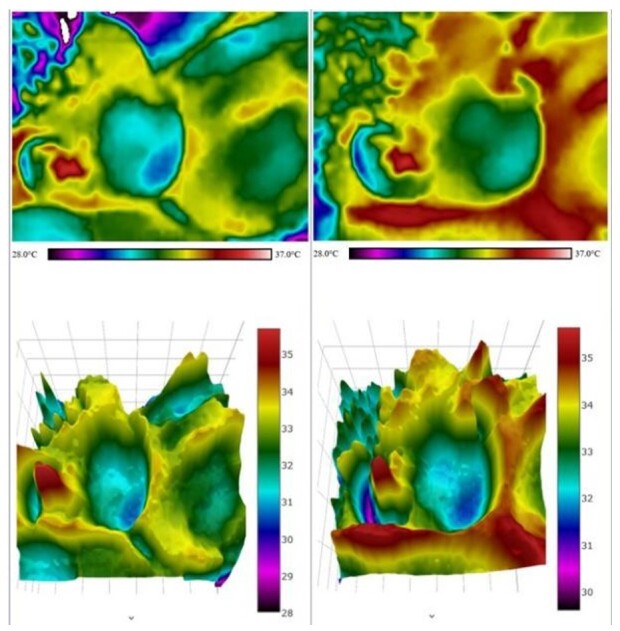
Image before and after lingual frenotomy

Images before and after LF, indicating that the temperature increased in the regions of temporal, masseter, and buccinator muscles (Figure 5).

## DISCUSSION

Ankyloglossia is adversely associated with successful breastfeeding and the mother’s well-being, with a 49.3% prevalence of breastfeeding difficulties and a mean nipple pain of 4.9 on the scale^(2)^. Systematic reviews considered the impact of LF surgical procedure on breastfeeding and concluded that it benefitted maternal pain^(25,26)^ and improves breastfeeding difficulties^(26)^. In the present study, maternal pain and breastfeeding difficulties was seen and significantly decreased after LF.

Breastfeeding assessment items that were unfavorable before LF and improved significantly after LF were also addressed in a study that verified the influence of LF on maternal observation and the infant’s position, latch, and suction^(14)^. The functional-anatomical assessment found significantly improved aspects after LF, demonstrating that this surgical procedure enables infants to have greater tongue mobility to perform orofacial functions – in this case, sucking.

Studies that used the same protocols as this research to diagnose ankyloglossia and assess breastfeeding indicate that the data on functional-anatomical aspects of ankyloglossia and unfavorable aspects of breastfeeding, especially regarding suction, corroborate the findings in this research^(27-29)^. The studies have increasingly approached suction assessment, but they still lack quantitative measures^(13)^, which is the objective of the present research. Differences in the IRT were expected and the value lies in quantifying the extent and quality of these changes. The qualitative analysis supports understanding of thermographic patterns even in the absence of statistically significant changes.

Comparative analyses conducted before and after LF demonstrated significant alterations in temporal, masseter, and buccinator skin temperature regions. The majority of infants exhibited elevated temperatures in the masseter and temporal areas. These regional temperature increases were attributed to the positive influence of lingual frenotomy on tongue movement, leading to enhanced engagement of the temporal and masseter muscles, which are responsible for jaw elevation. This phenomenon likely reflects a more balanced involvement of the muscles participating in the sucking activity, with the increased surface temperature may reflect greater regional blood flow due to improved muscle engagement during sucking.

Among other things, breast milk is extracted thanks to oscillations in atmospheric pressure caused by changes in intraoral volume resulting from mandible movements and tongue wave movements in the anterior-posterior direction^(10,11)^. This is in line with another ultrasound assessment of tongue movements during breastfeeding, demonstrating that babies with ankyloglossia exert greater nipple compression and that they perform longer bursts during sucking assessed using a pressure sensor^(30)^. It is expected that after LF there will be better grip, greater participation of tongue movements and jaw elevator muscles, contributing to better sucking^(9)^.

Furthermore, the surface electromyographic evaluation identified that sucking after LF required less activity of the masseter muscle, possibly demonstrating participation of this muscle with reduced effort^(14)^. These findings may not be directly correlated. The temperature changes reflect surface heat and not necessarily contraction intensity. Electromyography (EMG) measures electrical activity, which may decrease if efficiency improves.

Future investigations should explore the sucking function and facial thermal distribution of infants longitudinally to expand upon the findings presented in this study. Correlating thermographic analysis with sucking function would provide valuable insights into the relationship between these two factors.

This study has limitations: i) absence of a control group for comparison; ii) maternal knowledge about breastfeeding was not tested; iii) mothers' breasts were not evaluated; vi) the assessment instruments may not have been sensitive enough to detect latch problems or may persist regardless of sucking mechanics; iv) lack of blinding of the speech-language pathologist evaluator before and after.

Due to following the breastfeeding position suggested in the literature, it was not possible to perform IRT mages of the suprahyoid region and it was not possible to check ROI temperatures bilaterally. However, this is an innovative study, as no other similar studies were found using the same methodology with the possibility of visualizing areas with greater activation or musculoskeletal balance using infrared thermography. Caution is suggested in interpreting the findings because IRT s a recent evaluation and there are methodological limitations to assess suction.

## CONCLUSIONS

Thermographic analysis of the baby's face revealed increase in temperature in the regions of the temporal and masseter muscles during breastfeeding after LF.
